# Quantitative determination of histone methylation via fluorescence resonance energy transfer (FRET) technology in immortalized bovine mammary alveolar epithelial cells supplemented with methionine

**DOI:** 10.1371/journal.pone.0244135

**Published:** 2020-12-21

**Authors:** Fernanda Rosa, Johan S. Osorio

**Affiliations:** Department of Dairy and Food Sciences, South Dakota State University, Brookings, South Dakota, United States of America; University of Illinois, UNITED STATES

## Abstract

Methionine (Met) is an essential precursor of S-adenosylmethionine (SAM), which is the primary methyl donor required for biological processes such as DNA and histone methylation, which alter gene expression. In dairy cows, dietary Met has been observed to exert transcriptional alterations with beneficial effects on milk biosynthesis; however, the extent of these effects via SAM remains unknown. Therefore, we evaluated the effect of Met supply on histone methylation in lysine residues K9 and K27 in the histone tail H3 via a fluorescence resonance energy transfer (FRET) system in immortalized bovine mammary alveolar epithelial cells (MACT) incubated varying concentration of Met. The histone methylation data was complemented with global DNA methylation, cellular protein synthesis, and RT-qPCR analysis of genes related to Met cycle, DNA and histone methylation, AA transporters, and protein synthesis. The histone methylation data was performed on MACT cells seeded at 30,000 cells/well in 96-well plates 24 h prior to transfection. The transfections of FRET gene reporter plasmids H3K9 and H3K27 was performed with 0.3 μL/well of Lipofectamine® 3000 and 50 ng of plasmid DNA per well. At 24 h post-transfection, cells were treated with 0, 125, 250, and 500 μM of Met, and quantification of histone methylation was performed at 0, 12, and 24 h post-treatment as well as cell viability at 24 h using CellProfiler software. An inverted microscope for live imagining (EVOS^®^ FL Auto) equipped with a motorized scanning stage, and an environment-controlled chamber at 37˚C and 5.0% of CO_2_ was used to take 4 pictures/well at 4x magnification. A more defined response on histone methylation was observed in H3K9 than H3K27 to Met supply, where maximal histone methylation in H3K9 was observed with 125 μM of Met. This greater histone methylation in H3K9 at 125 μM was accompanied by greater cellular protein concentration. The linear increase in Met supply causes a linear decrease in global DNA methylation, while linearly upregulating genes related to the Met cycle (i.e., *MAT1A*, *PEMT*, *SAHH*, and *MTR*). The histone methylation data suggest that, to some extent, methyl-donors such as Met may affect the methylation sites, H3K9 and H3K27, and consequently causing a different epigenetic alteration. In the context of the dairy cow, further refinement to this FRET assay to study histone methylation could lead to establishing novel potential mechanisms of how dietary methyl donors may control the structural conformation of the bovine genome and, by extension, gene expression.

## Introduction

Methionine (Met) and lysine [1] are the most limiting amino acids (AA) in a wide range of diets for dairy cows [2]. Among the various biological functions associated with Met, besides milk protein synthesis, gene expression regulation in dairy cows has become more evident in recent years [3, 4]. Some of the specific mechanisms in ruminants for this effect have been previously discussed as DNA methylation [5], transcription factors [6], and histone methylation [7]. However, the extent of Met effects through these mechanisms in transition dairy cows remains to be elucidated. Histone modifications such as methylation can considerably alter the available regions or information contained in the DNA that can be transcribed into mRNA and subsequent translation into proteins. Thus, this can potentially change the metabolism, health, and performance of dairy cows. Until now, the limited amount of data on bovine histone methylation has focused on mastitis [8, 9], but based on the substantial effects of Met supplementation on DNA methylation in transition dairy cows [10], it is conceivable that this nutrient can also have a significant impact on histone modifications through methylation.

Histones are proteins (i.e., H1, H2A, H2B, H3, and H4) found in the cell nuclei, and are responsible for packing and arranging the DNA by acting as spools around which the DNA winds and they play a significant role in gene regulation. The functional structure of histones can be modified through several biological processes such as acetylation, phosphorylation, and methylation, among others [11]. Reviewed by Greer and Shi [12], methylation of histones can occur in residues of lysine, arginine, and histidine. However, lysine is the residue that can be mono (me1), di(me2), or tri(me3) methylated on their amine group. Methylation is most commonly observed on lysine residues of histone tails H3 and H4 [13]. The lysine sites for methylation have been extensively studied, including H3 lysine 9 (H3K9), and H3 lysine 27 (H3K27) [8, 14].

The S-adenosylmethionine (SAM) is the universal methyl-donor for the multiple types of methyltransferase reactions in the cell [15], and among these reactions are the DNA methylation and histone methylation. Since Met is the primary source for SAM synthesis in the Met cycle [16], methylation activity can likely be altered by Met availability. Rijnkels, Freeman-Zadrowski [17] demonstrated that histone methylation regulates the chromatin organization in mouse lactating mammary gland, leading to the regulation of essential milk protein genes as caseins. The latter was related to di-methylation of the histone H3 at lysine 4 (H3K4me2), leading to an open chromatin during lactation with marks for the casein gene promoter region.

The potential of using relatively new molecular technologies such as gene reporter technology based on fluorescent proteins in dairy cattle molecular biology has been discussed previously [18]. Lin and collaborators developed a dual-fluorescent protein system [i.e., fluorescent resonance energy transfer (FRET)] to determine histone methylation at the methylation sites of interest K9 and K27 [19]. This fluorescent protein system allows the visualization of histone methylation in mammalian cells in real-time through fluorescent microscopy; however, this method has never been used in evaluating the Met effects on histone methylation in bovine mammary cells.

Based on the known regulation of histone methylation by Met metabolism in humans [20], and the findings in dairy cows associating dietary Met with changes in gene expression [4, 21], and global DNA methylation [10], we hypothesized that incubating bovine mammary cells (MACT) cells with incremental Met concentrations in the media will likely modify histone and DNA methylation as well as genes related to Met cycle, DNA and histone methylation, AA transporters, and protein synthesis. Therefore, we aimed to evaluate the effects Met supply on histone methylation in lysine residues K9 and K27 in the histone tail H3 via a FRET system in immortalized bovine mammary alveolar epithelial cells MACT [22, 23] incubated at 0, 125, 250, and 500 μM of Met.

## Materials and methods

### Cell culture and plasmid purification

The cell culture and plasmid purification were performed as previously described in Osorio and Bionaz [24]. In this study, the MACT cells were generously provided by Massimo Bionaz, Department of Animal and Rangeland Sciences, Oregon State University. Cells were cultivated in a high-glucose Dulbecco modified Eagle's medium (DMEM) with sodium pyruvate (Cat#10013CV, Corning Cellgro, Manassas, VA) while treatments were applied using a reduced serum medium Opti-MEM® (Cat#11058021; Life Technologies, Grand Island, NY). The DMEM medium was supplemented with 10% fetal bovine serum (Cat# 1500–500; Seradigm, Radnor, PA), penicillin/streptomycin (10 mL/L, Cat# 97063–708; Amresco, Solon, OH), and Fungizone® Antimycotic (3μL/mL, Cat# 15290–018; Life Technologies, Grand Island, NY). The histone methylation reporters, pcDNA3-K27, and pcDNA3-K9 plasmids utilized in this study (Addgene plasmid#22865, and plasmid #22866, respectively) were cloned using the DH5alpha competent cells (Cat# 18265017; Life Technologies, Grand Island, NY) after incubation with Luria broth (50 mL) overnight, and subsequently isolated and purified using the GeneJet Plasmid Miniprep Kit (Cat# K0481; Life Technologies, Grand Island, NY). Briefly, bacterial culture (~50 mL) post-selected via Ampicillin was centrifuged for 10 min at 5000 × *g*. After centrifugation, pelleted cells were resuspended in 2 mL Resuspension Solution, followed by addition of 2 mL of Lysis Solution, and incubated for 3 min at room temperature. After incubation, 2 mL of Neutralization Solution added, followed by 0.5 mL of endotoxin binding reagent and incubated for 5 min at room temperature. After incubation, 3 mL of 96% ethanol was added, and the solution was centrifuged for 40 min at 4,500 × *g* to pellet cell debris and chromosomal DNA. After centrifugation, the supernatant (~6–7 mL) was transferred to a new 15 mL tube and mixed with 3 mL of 96% ethanol. This solution was passed through a silica-based membrane column via centrifugation for 3 min at 2000 × *g*, then the plasmids bound to the membrane were purified with two different wash solutions (I and II). Finally, the purified plasmids were eluted with 350μL of Elution Buffer. Concentration and purity of plasmids were assessed with a Nanodrop ND-1000 spectrophotometer (Nanodrop Technologies, Wilmington, DE), for which the 260/280 ratio was above 1.9.

### Transfection and treatments

In order to evaluate the effects of Met on histone methylation, MACT cells were transfected with a plasmid DNA that encodes for a modified peptide substrate corresponding to the methylation site of interest within histone tail in H3, and capable of emitting a FRET type signal using cyan and yellow fluorescent proteins. MACT cells were seeded at 30,000/well in 96-well plates 24 h prior to transfection. The transfection reagent used was Lipofectamine® 3000 (Lipo3; Cat# L3000001; Life Technologies) at a concentration of 0.3μL/well. The final volume per well used was 100μL for all transfection conditions and 50 ng of plasmid DNA per well. The treatments were applied 24 h post-transfection, and each treatment was performed in triplicates in each plate as follow: L-Met (Cat# 63683, Acros Organic) was dissolved in Opti-MEM® to a final stock solution of 50mM, and subsequent dilutions with Opti-MEM® were performed to get the final working solutions: 125μM, 250μM, and 500μM of Met. These treatments were applied to a total of 4 plates performed on different days. In each plate, one of the treatments was seeded cells only incubated with 100 μL of Opti-MEM® to be used as a positive control for viability, and additional treatment of cells incubated with 100 μL of 70% molecular ethanol (diluted with Opti-MEM®) for 20 min at 24 h post-treatment to be used as a negative control for viability.

### Imaging fluorescence analysis

After the treatments were applied, an automated epifluorescent microscope for live imaging (EVOS FL Auto Imaging System, Cat# AMF4300, Thermo Fisher Scientific Inc, Bothell, WA) equipped with a motorized X-Y scanning stage, LED light engine, and environmental chamber to maintain cells at 37˚C and 5.0% of CO_2_ (Invitrogen™ EVOS™ Onstage Incubator, Cat# AMC1000) was used to take 4 pictures/well at 0, 12, and 24 h post-treatment. For the histone methylation analysis, each picture was an overlay of cyan fluorescent protein [CFP, excitation (nm) 445/45, and emission (nm) 510/42, EVOS™ LED cube #AMEP4653, Thermo Fisher Scientific Inc, Bothell, WA] and a fluorescence resonance energy transfer-FRET [CFP-YFP, excitation (nm) 445/45, and emission (nm) 542/27, EVOS™ LED cube #AMEP4669, Thermo Fisher Scientific Inc, Bothell, WA] detected with the corresponding fluorescent light.

The open-source software CellProfiler (Kamentsky et al., 2011) was used to analyze each picture to quantify cell number and intensity of all cells in each picture. The total intensity of cells per image was calculated with the CellProfiler based on cells that expressed both CFP and CFP-YFP fluorescent intensities (i.e., colocalization). Then, a ratio of total intensity of CFP-YFP over CFP was calculated to obtain a YFP signal, which is the signal associated with histone methylation. Live and Dead cells were measured at 24 h post-treatment. Dead cells were measured using a nuclear stain (Propidium Iodide®, Cat# R37108; Life Technologies) over a red filter (Texas Red, excitation at 585/29nm, and emission at 624/40nm, EVOS™ LED cube, Cat# AMEP4655). While live cells were measured using a Hoechst stain (NucBlue® Live ReadyProbes® Reagent, Life Technologies) over a blue filter (DAPI, excitation at 357/44nm, and emission at 447/60nm, EVOS™ LED cube, Cat# AMEP465). Cell viability was calculated by colocalizing the number of dead cells over the number of blue cells in a given picture and multiplying the result by 100 [24].

### DNA isolation, global DNA methylation, and total protein assay

MACT-cells seeded at 1.06x10^6^ density in 6-well plates were incubated with the methionine doses (125μM, 250μM, and 500μM) for 24h. At 24 h post-treatment, cells were detached with trypsin-EDTA (Cat#25300054; Life Technologies) for further DNA and total protein analysis. The DNA isolation and the global DNA methylation assays were performed as previously published [5] with some modifications. Briefly, the cell pellet was lysed using proteinase K, loaded to the DNeasy Mini spin column (Blood and Tissue DNeasy Kit, Qiagen, Germany), DNA was selectively bound to the membrane, and contaminants were washed and finally eluted in water. The total protein concentration was determined using the BCA Assay Kit (Cat# 23227; Thermo Scientific).

Global DNA 5-methylcytosine (5-mC) was quantified using the 5-mC ELISA DNA kit (Zymo Research). The kit works with a unique anti-5-mC monoclonal antibody that is sensitive and specific for 5-mC across a wide range of starting DNA amount. The 5-mC percentage was quantified based on a standard curve generated by combining the positive (100%) and negative controls (0%). A total of 100 ng of DNA were mixed with a 5-mC coating buffer, denatured at 98°C for 5 min, placed on ice immediately, and then incubated in duplicate in a 96-well plate at 37°C for 1 h. The coating buffer and samples were discarded, washed 3× with 200 μL of 5-mC buffer, and incubated at 37°C for 30 min with 5-mC buffer. After discarding the contents, 100 μL of the antibody mix containing anti-5-mC, secondary antibody, and 5-mC buffer was added and incubated at 37°C for 1 h. After discarding the contents, the washing step was performed as before, with 100 μL of horseradish peroxidase-developer added and incubated at room temperature for 1 h. Absorbance at 405 and 450 nm was measured using a plate reader (Cary 50, Varian Inc., Walnut Creek, CA).

### RNA isolation, cDNA synthesis, primer design, and evaluation and quantitative PCR

The RNA isolation from cell pellets was carried out using the RNeasy Plus Mini Kit (Cat#74134; Qiagen), following the manufacturer’s instructions with some modifications. Briefly, prior to the RNA isolation, the cell pellets (~1.06 x 10^6^cells) were homogenized using a beadbeater (Cat# 607; Biospec Products, Inc.) while immersed in 1 mL of TRIzol Reagent (Cat# 15596018, Ambion, Carlsbad, CA, USA). After homogenization, 200μl of chloroform (EMD Millipore, Germany, Cat. No. CX1055-6) was added in order to isolate the RNA from the organic phase. The RNA quantity (368 ± 143.09 ng/μL; mean ± SD), and purity as 260/280 ratio and 260/230 ratio (2.03 ± 0.02; and 1.81 ± 0.27, respectively) were determined using Nanodrop ND-1000 (NanoDrop Technologies, Rockland, DE). Details of cDNA synthesis, primer design, and evaluation, as well as quantitative PCR, are presented in the S1 File. The selected target genes play important roles in the Met cycle [Methionine adenosyltransferase 1A (*MAT1A*), phosphatidylethanolamine N-methyltransferease (*PEMT*), S-adenosylhomocysteine hydrolase (*SAHH*), and 5-methyltetrahydrofolate-homocysteine methyltransferase (*MTR*)], histone methylation [SET domain bifurcated histone lysine methyltransferase (*SETDB1*), euchromatic histone lysine methyltransferase (*EHMT2*), and suppressor of variegation 3–9 homolog 1 (*SUV39H1*)], DNA methylation [DNA methyltransferase 3 alpha (*DNMT3A*) and DNA methyltransferase 1 (*DNMT1*)], AA transporters [solute carrier family 3 member 2 (*SLC3A2*), solute carrier family 1 member 5 (*SLC1A5*), solute carrier family 38 member 2 (*SLC38A2*), solute carrier family 38 member 9 (*SLC38A9*)], and protein synthesis [mammalian target of rapamycin (*MTOR*), ribosomal protein S6 kinase B1 (*RPS6KB1*)].

### Statistical analysis

The coefficient of variation (CV %) was calculated based on data from 4 pictures per well, and data from each picture with the farthest deviation from the mean per well was removed if the CV % was > 5%. Then, the mean per well was used for statistical analysis. The relative histone methylation in H3K9 and H3K27 was analyzed as repeated measures using the PROC MIXED of SAS (SAS Institute Inc., Cary, NC) with plate, Met concentration, time (i.e., 0, 12, and 24 h post-treatment), and their interaction as the fixed effects in the model. The well nested within plate and treatment was used as the random effect. The relative histone methylation measured at 0 h post-treatment was used as a covariate for the repeated measures analysis. Data measured at 24h post-treatment, including global DNA methylation, gene expression, total protein concentration, and cell viability, the plate and Met concentration were the only variables considered as a fixed effect in the model. In the histone methylation data, two observations in two different plates were considered outliers and removed from the analysis. The *P*-value significance was adjusted with the Tukey test for mean separation. Statistical significance and tendencies were declared at *P* ≤ 0.05 and 0.05 ≤ *P* ≤ 0.10, respectively, for all comparisons.

## Results

### Relative histone methylation and viability

**Fig 1:**
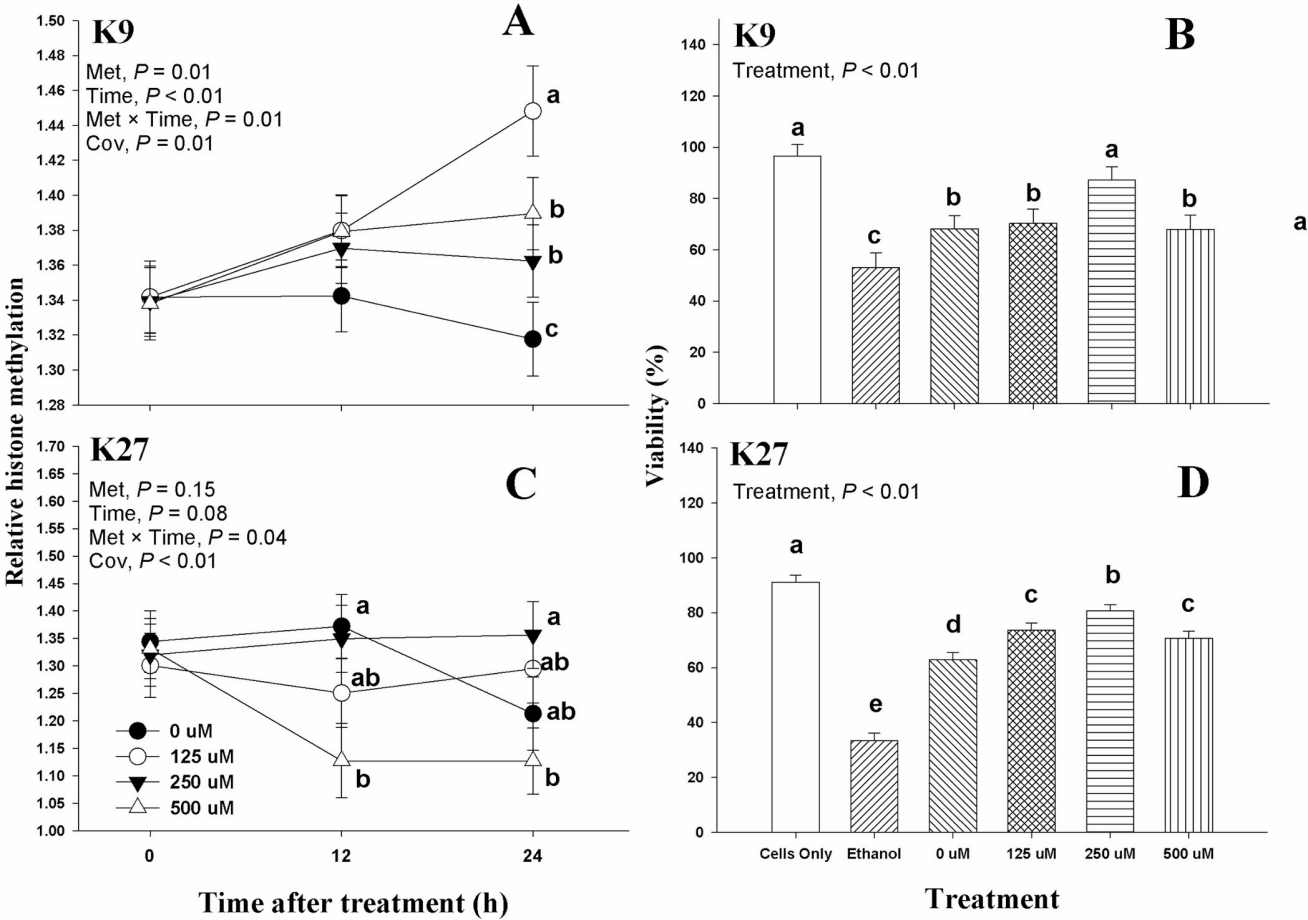
Effect of supplemental Met at 0, 125, 250, and 500 μM on relative histone methylation and viability in bovine mammary alveolar epithelial cells (MACT). *A*: relative histone methylation in lysine residue 9 (K9) in the tail of histone 3 (H3) at 0, 12, and 24 h post-treatment; *B*: viability of MACT cells at 24 h post-treatment with 0, 125, 250, 500 μM of Met as well as cells only (positive control) and ethanol (negative control); *C*: relative histone methylation in lysine residue 27 (K27) in the tail of H3 at 0, 12, 24 h post-treatment; *D*: viability of MACT cells at 24 h post-treatment with 0, 125, 250, 500 μM of Met as well as cells only (positive control) and ethanol (negative control). Values are means, with standard errors represented by vertical bars.

Main effects and interactions of Met supplementation on histone methylation at 0, 12, and 24 h post-treatment and viability at 24 h post-treatment are shown in Fig 1. There was an interaction (*P* ≤ 0.04) of Met supplementation and time (Met × Time) in K9 and K27 (Fig 1A and 1C). The Met × Time in K9 was mainly associated with different histone methylation at 24 h post-treatment, where the 125 Met treatment produced the greatest (*P* < 0.01) histone methylation, while the 0 Met treatment had the lowest (*P* < 0.01) histone methylation (Fig 1A). In the case of K27, at 12 h post-treatment, histone methylation levels were greater (*P* < 0.01) in 0 and 250 Met in comparison to 500 Met (Fig 1C). Then, at 24 h post-treatment, 250 Met treatment remained greater (*P* = 0.01) than 500 Met, while 0 and 125 Met treatments had intermediate levels of histone methylation. A treatment effect (*P* < 0.01) on viability at 24 h post-treatment in K9 and K27 (Fig 1B and 1D). A greater viability was observed in K9 in cells only (i.e., cells were neither transfected nor treated with Met) and the 250 Met treatment (96.5 and 87.1%, respectively), while the cells only treatment had the highest (91.1%) viability in K27. In both K9 and K27, cells treated with ethanol had the lowest viability (53.1% and 33.4%, respectively). In terms of the Met treatments, the 250 Met treatment had the highest viability in both K9 and K27.

**Fig 2:**
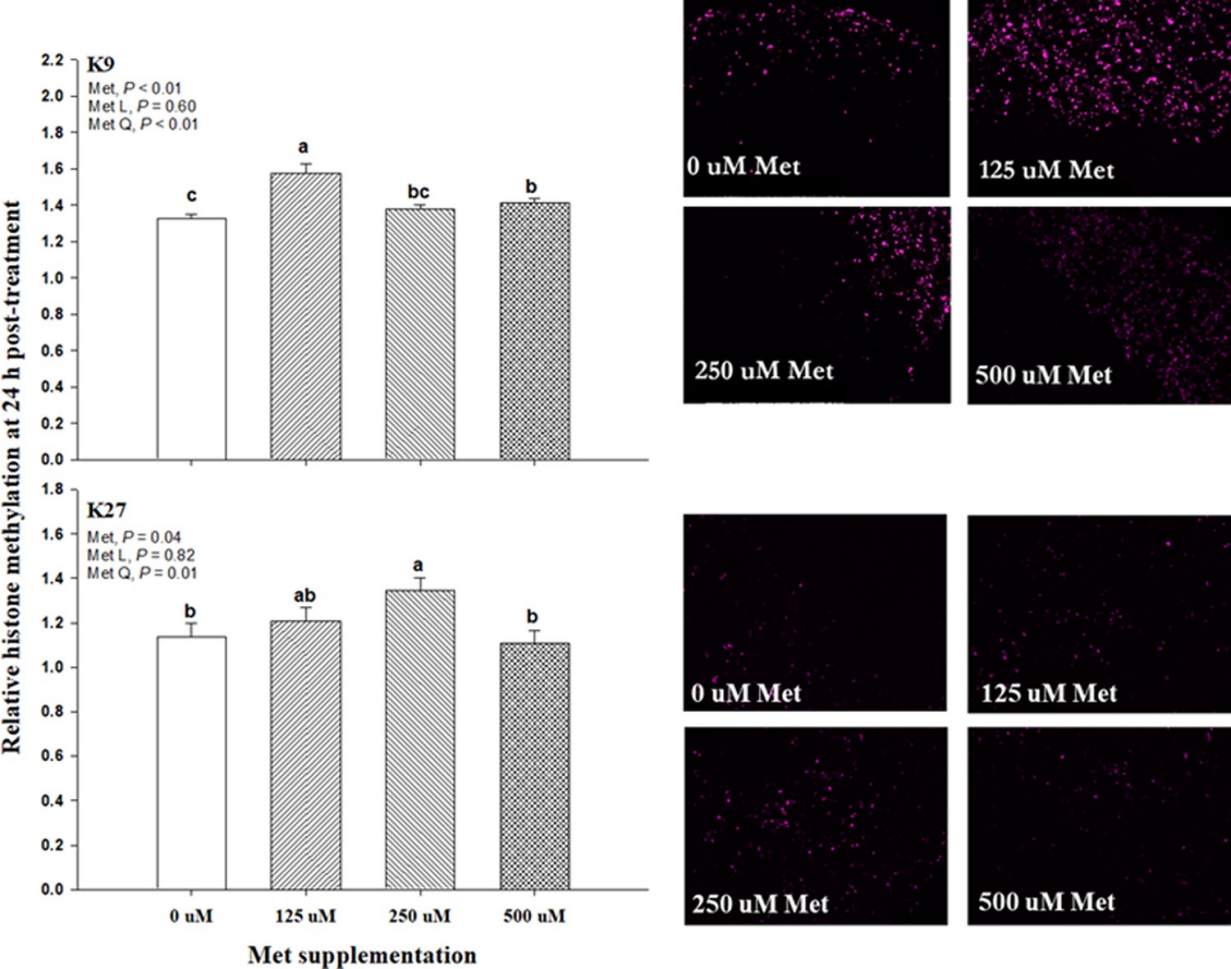
Effect of supplemental Met at 0, 125, 250, and 500 μM on relative histone methylation at 24 h post-treatment in bovine mammary alveolar epithelial cells (MACT). Relative histone methylation in lysine residue 9 (K9) or lysine residue 27 (K27) was measured through a fluorescent resonance energy transfer. A priori orthogonal contrasts were analyzed in order to determine linear (L) and quadratic (Q) effects over Met supplementation. Values are means, with standard errors represented by vertical bars.

Histone methylation analysis at 24 h post-treatment is shown in Fig 2. There was a treatment effect (*P* ≤ 0.04) in K9 and k27, and this effect was reflected on a consistent quadratic effect (*P* ≤ 0.01) in both K9 and K27 over the Met treatments. However, the greatest histone methylation in K9 and K27 was at 125 μM and 250 μM, respectively.

### Methionine effect on DNA methylation and total protein

**Fig 3:**
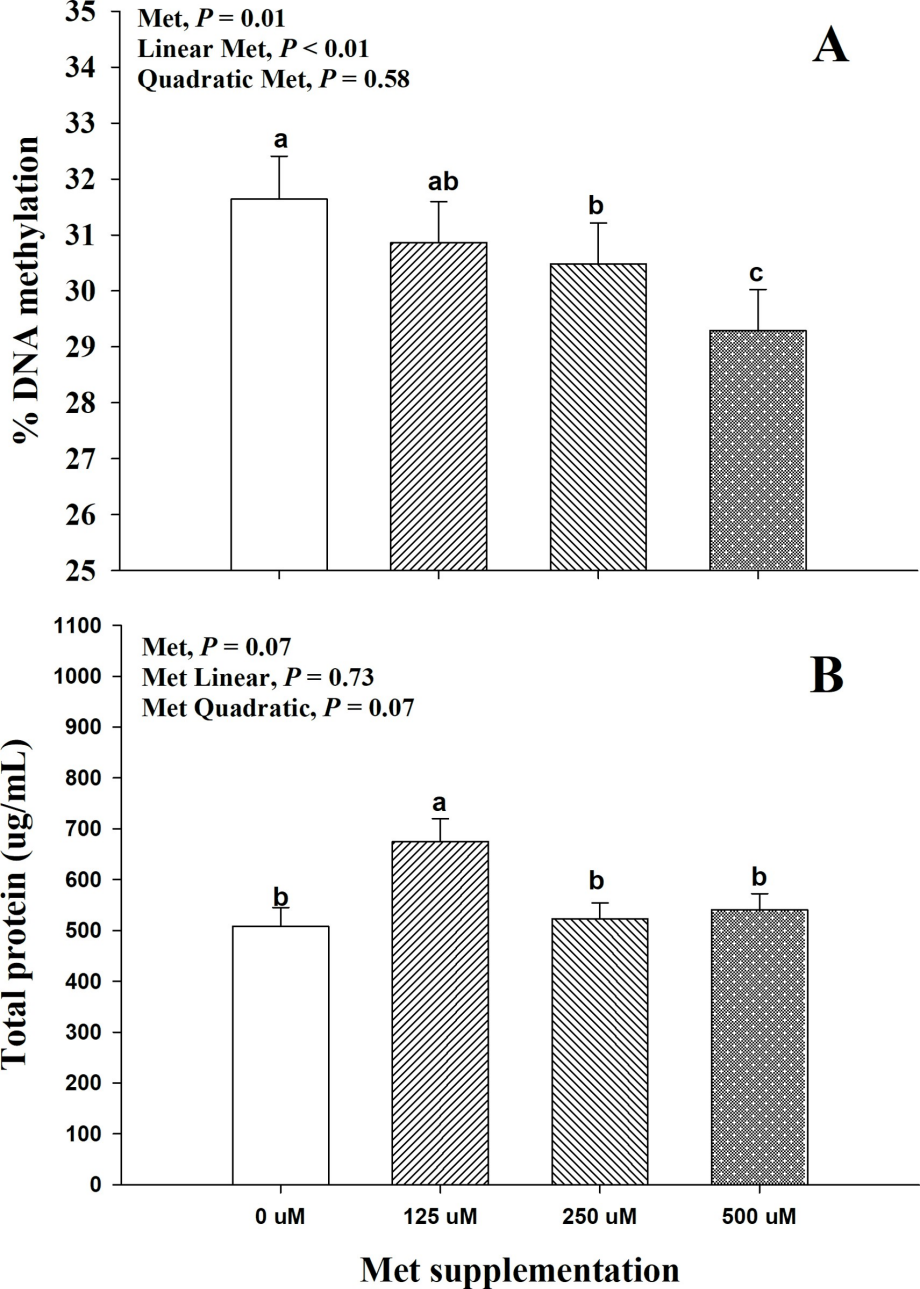
Effect of supplemental Met at 0, 125, 250, and 500 μM on (A) DNA methylation and (B) total protein at 24 h post-treatment in bovine mammary alveolar epithelial cells (MACT). A priori orthogonal contrasts were analyzed in order to determine linear (L) and quadratic (Q) effects over Met supplementation. Values are means, with standard errors represented by vertical bars.

Main effects and interactions for Met supplementation on global DNA methylation and total protein concentration at 24 h post-treatment are shown in Fig 3. Global DNA methylation was affected by methionine supplementation (*P* = 0.01), which was reflected in a linear (*P* < 0.01) decreased in global DNA methylation as Met supplementation increased from 0 to 500 μM (Fig 3A). A trend (*P* = 0.07) for a Met effect on total protein concentration was observed on Met supplementation, and it was reflected in a quadratic effect (*P* = 0.07) with the highest total protein concentration at 125 μM (Fig 3B).

### Methionine effect on gene expression

#### The Met cycle

**Fig 4:**
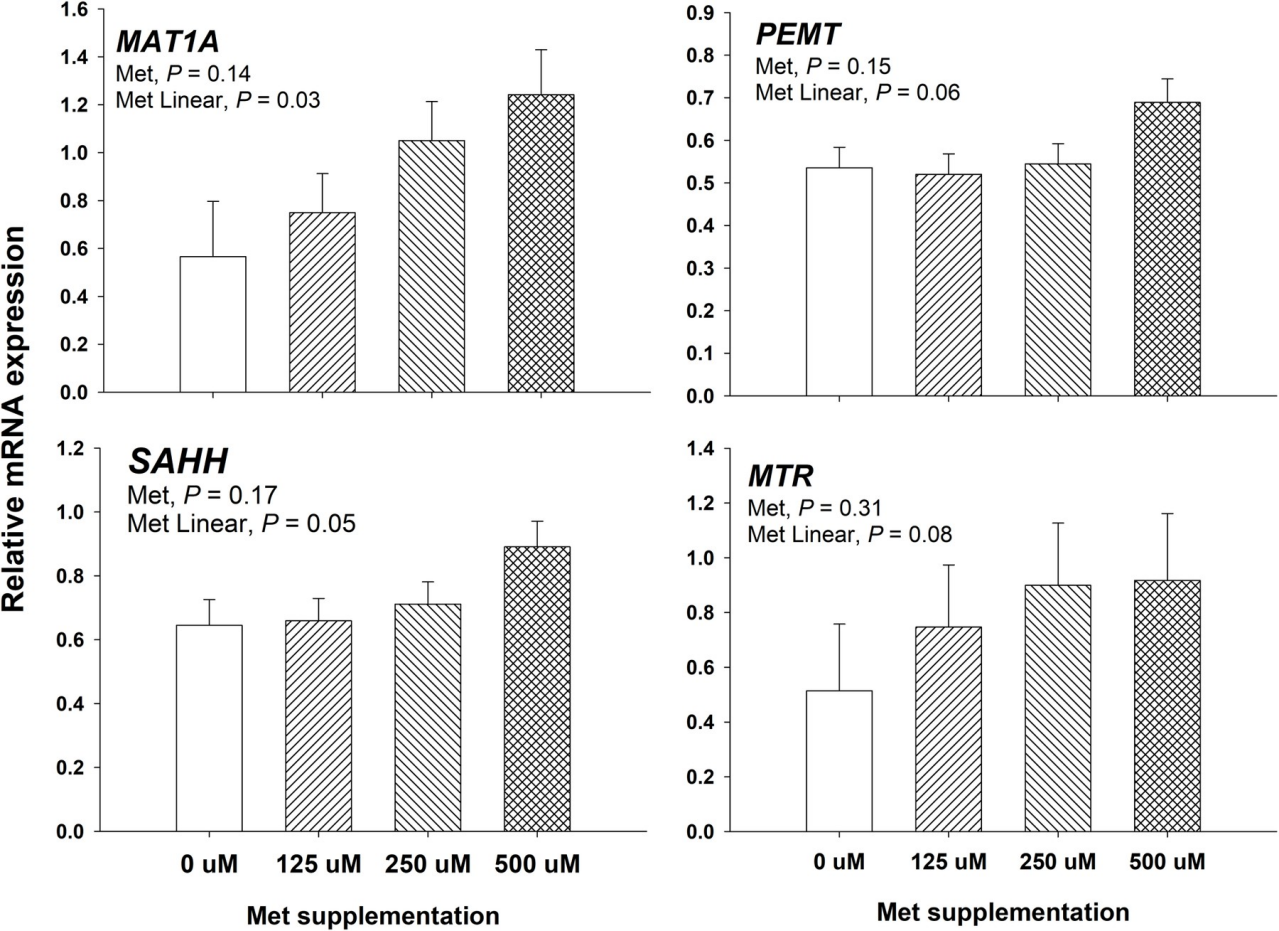
Effect of supplemental Met at 0, 125, 250, and 500 μM on mRNA expression of genes related to the Met cycle. Targeted genes for the Met cycle included Met adenosyltransferase 1A (*MAT1A*), phosphatidylethanolamine methyltransferase (*PEMT*), s-adenosylhomocysteine hydrolase (*SAHH*), and 5-methyltetrahydrofolate-homocysteine methyltransferase (*MTR*) at 24 h post-treatment in bovine mammary alveolar epithelial cells (MACT). A priori orthogonal contrasts were analyzed in order to determine linear (L) and quadratic (Q) effects over Met supplementation. Values are means, with standard errors represented by vertical bars.

Main effect for Met supplementation on genes associated with the Met cycle is shown in Fig 4. An overall effect (*P* ≥ 0.14) of Met supplementation was not observed on the genes related to the Met cycle. However, a linear upregulation (*P* ≤ 0.05) of the genes *MAT1A* and *SAHH* was observed as Met supplementation was increased from 0 to 500 μM. Similarly to *MAT1A* and *SAHH*, a trend for an upregulation on the mRNA expression of the genes *PEMT* (*P* = 0.06) and *MTR* (*P* = 0.08) was observed as Met supplementation was increased from 0 to 500 μM.

#### Histone and DNA methylation

Main effect for Met supplementation on genes associated with histone and DNA methylation is shown in Table 1. The *SUV39H1* was the only gene with an overall effect (*P* = 0.04) for Met supplementation, and to a lesser extent *DNMT1* (*P* = 0.06) and SETDB1 (*P* = 0.07) had a trend for a Met effect. Then, while the Met effect on *SUV39H1* and *DNMT1* was described by a positive quadratic effect (*P* ≤ 0.03), the Met effect on *SETDB1* as main associated with a linear increase as Met supplementation increased from 0 to 500 μM (Table 1). In contrast to *SETDB1*, *DNMT3A* mRNA expression tended (*P* = 0.07) to be downregulated when Met supplementation was increased from 0 to 500 μM (*P* (Table 1)).

**Table 1:** Effect of supplemental Met at 0, 125, 250, and 500 μM on mRNA expression of genes related to DNA and histone methylation, AA transporters, and protein synthesis at 24 h post-treatment in bovine mammary alveolar epithelial cells (MACT).

Gene^1^	Met supplementation (μM)				SEM^3^	*P*-value^2^		
	0	125	250	500		Met	L	Q
Histone methyltransferase								
*SETDB1*	-0.61	-0.66	-0.30	-0.29	0.17	0.07	0.03	0.76
*EHMT2*	-1.58	-1.42	-1.45	-0.83	0.31	0.33	0.11	0.38
*SUV39H1*	-1.05	-1.46	-1.11	-0.93	0.17	0.04	0.18	0.03
DNA methyltransferase								
*DNMT3A*	-0.14	-0.58	-0.46	-0.62	0.41	0.14	0.07	0.31
*DNMT1*	0.23	-0.36	-0.23	0.71	0.40	0.06	0.19	0.01
AA transporters								
*SLC3A2*	-0.80	-0.61	-0.43	-0.45	0.15	0.28	0.08	0.48
*SLC1A5*	-0.80	-0.77	-0.49	-0.46	0.26	0.49	0.18	0.99
*SLC38A2*	1.07	0.83	0.75	0.69	0.26	0.01	<0.01	0.21
*SLC38A9*	-0.08	0.004	0.35	0.42	0.32	0.11	0.03	0.95
Protein synthesis								
*MTOR*	-0.19	-0.46	-0.19	-0.45	0.29	0.11	0.24	0.92
*RPS6KB1*	1.14	0.79	1.09	1.14	0.28	0.11	0.54	0.09

^1^Targeted genes for DNA methylation [DNA methyltransferase 3 alpha (*DNMT3A*) and DNA methyltransferase 1 (*DNMT1*)], histone methylation [SET domain bifurcated histone lysine methyltransferase (*SETDB1*), euchromatic histone lysine methyltransferase (*EHMT2*), and suppressor of variegation 3–9 homolog 1 (*SUV39H1*)], AA transporters [solute carrier family 3 member 2 (*SLC3A2*), solute carrier family 1 member 5 (*SLC1A5*), solute carrier family 38 member 2 (*SLC38A2*), solute carrier family 38 member 9 (*SLC38A9*)], and protein synthesis [mammalian target of rapamycin (*MTOR*), ribosomal protein S6 kinase B1 (*RPS6KB1*)] at 24 h post-treatment in MACT cells.

^2^SP-values represent the probability of statistical significance for the overall Met effect and a priori orthogonal contrasts to determine linear (L) and quadratic (Q) effects over Met supplementation.

^3^Largest SEM.

#### AA transporters and protein synthesis

Main effect for Met supplementation on genes associated with AA transporters and protein synthesis is shown in Table 1. The *SLC38A2* gene was the only gene with a Met effect (*P* = 0.01), and this was attributed to a linear downregulation in its expression as Met supplementation increased from 0 to 500 μM. In contrast to *SLC38A2*, *SLC38A9* had a linear upregulation (*P* = 0.03) as Met supplementation increased from 0 to 500 μM, and a trend (*P* = 0.08) for a similar effect was observed in *SLC3A2*. A trend (*P* = 0.09) for a positive quadratic effect was observed in *RPS6KB1*. The Met supplementation did not affect (*P* ≥ 0.11) the expression of *SLC1A5* and *MTOR*.

## Discussion

The application of fluorescent proteins for tracking single molecules in live cells or the whole organism has been vastly exploited and has impacted the fields of biochemistry, biotechnology, and cell biology [18]. In animal sciences, fluorescent proteins have been mainly used as molecular markers that can cast a signal based on cell type identification, or presence of specific proteins in cell culture and tissue explants (e.g., immunochemistry). Another application for fluorescent proteins is gene reporter technology, which has been used to monitor the activation of transcription factors by fusing the appropriate DNA coding for a promoter region, including the response element of the target gene with the DNA sequence coding for a fluorescent protein (or reporter gene). Luciferase is by far the most-used gene reporter technology in nutrigenomic studies in dairy cows but has been used to study other cellular events (e.g., cell signaling and milk protein expression) in bovine cells [25]. In contrast to luciferase reported assay, the FRET technology is a less known technique, but with a growing interest in biomedical science and biotechnology fields [26]. To the authors' knowledge, the use of a FRET system to track histone methylation has never been tested before in bovine cells. The FRET system is based on the ability to transfer energy between two fluorescent proteins from a donor to an acceptor depending on their spatial proximity. The closer the two fluorescent proteins are, the more fluorescent emission will be detected from the acceptor, which in the current study describes greater histone methylation [27]. The FRET system utilized in this study was designed by Lin and collaborators [19], and it comprises of a histone-derived peptide sandwiched between a pair of fluorescence resonance energy transfer-capable green fluorescent protein (GFP) mutants, cyan fluorescent protein (donor) and a yellow fluorescent protein (acceptor). This is the first study in a bovine cell model utilizing FRET and fluorescent microscopy to derive a signal on histone methylation via Met supplementation. There is a potential on developing this technology to advance our understanding of how methyl donors such as Met can affect the available genome via epigenetics in dairy cows.

### Methionine cycle and protein synthesis

Methionine is an essential amino acid, and its beneficial effects on the performance of livestock animals have been extensively studied. In dairy cattle, such beneficial effects have been commonly attributed to a low abundance of this amino acid in forages and feedstuff normally used in dairy cattle nutrition [2]. Therefore, supplementation of rumen-protected Met to transition dairy cows has consistently improved milk yield [28–30] and milk composition [28–31]. Although such a response in performance has been accompanied by an evident transcriptomic alteration [21], the extent of the impact of these molecular alterations on the final performance of dairy cows being supplemented with Met remains unknown. In the current study, we observed a consistent linear upregulation in genes related to the Met cycle (Fig 4). This is in agreement with previous *in vivo* studies where hepatic gene expression of *MAT1A*, *PEMT*, and *SAHH* was upregulated in transition dairy cows supplemented with methionine [4, 32]. Taken together, these data suggest that genes related to the Met cycle are highly responsive to changes in the availability of this AA in either *in vivo* or *in vitro* models.

The transport of AA into epithelial cells in the mammary gland is one of the major limitations for milk protein synthesis. This is predicated on the known intermediary metabolism and utilization of AA between the duodenum and the mammary gland, which accounts for the decreased efficiency of the transfer of absorbed AA into milk protein [33]. Therefore, AA transporters play a key role in the procurement of AA for milk biosynthesis in the mammary gland. Among the AA transporters measured in this study, *SLC38A2* and *SLC38A9* were the most responsive to varying concentrations of Met in the media (Table 1), where mRNA transcription of *SLC38A2* and *SLC38A9* linearly decreased and increased, respectively. Similar to our results, Dong, Zhou [34] observed a decreased mRNA expression of *SLC38A2* in MATC cells as Met was increased from 60 to 87 μg/mL of Met (or 402.1 to 583.1 μM of Met) in the media. This consistent effect on *SLC38A2* by Met in MACT cells indicates an underlying mechanism as explained by Dong, Zhou [34], whereby an increased supply of Met could produce a negative feedback on the uptake of AA when EAA such Met are balanced. In contrast to our results, Ma and collaborators [35] observed an upregulation in the neutral AA transporter, *SLC38A2*, in postpartal mammary tissue of transition dairy cows fed a rumen-protected Met supplement. Discrepancies in *SLC38A2* response to Met between *in vitro* and mammary tissue could be related to the Met concentration, as higher Met concentrations were tested *in vitro* (i.e., maximal Met at 583.1 and 500 μM in [34] and the current study, respectively) in comparison to in vivo [35], where the blood plasma concentration of Met in peripartal dairy cows was ~11 μM. The upregulation in *SLC38A9* product of Met supplementation in the current study was similar to the increase in mRNA expression observed in mammary tissue [35], while contrary to the downregulation in *SLC38A9* observed by Dong, Zhou [34] in MACT cells. A major difference between the current study and Dong, Zhou [34] was that the latter maintained ideal AA ratios (i.e., Thr:Phe 1.05:1; Lys:Thr 1.8:1; Lys:His 2.38:1; Lys:Val 1.23:1) while increasing the concentration of Met in the media. Then, this could serve as a plausible explanation for differences in mRNA expression of *SLC38A9*. A similar trend for upregulation of *SLC3A2* in the current study and Dong, Zhou [34] suggest a stimulation of this AA transporter when Met is supplied in the media; however, *SLC3A2* seemed to be less responsive to Met in vivo [35].

The mammalian target of rapamycin (mTOR) is essential for the initiation of protein synthesis. Although some AA, such as leucine, are well known for stimulating the activity of mTOR, Met has been observed to have inconsistent effects on mTOR activity, in terms of the ratio of p-mTOR:mTOR [34–37]. Similar to our results, Nan, Bu [36] observed no effects on *MTOR* mRNA expression by Met supplementation. This suggests that Met may influence alternative metabolic routes to improve milk protein synthesis, such as upregulation of AA and glucose transporters, insulin signaling, and AA tRNA-synthases, as observed by Ma, Batistel [35]. However, it is noteworthy to highlight the concomitant increased in total cellular protein (Fig 3B) and H3K9 methylation at 125 uM Met supplementation (Fig 2), suggesting a potential mechanism by which an adequate level of H3K9 methylation will promote an optimal DNA structure to enhance transcription. Unfortunately, this current study does not include sufficient additional data to provide further explanations on this mechanism. However, it is plausible that H3K9 methylation could be linked to alterations in the protein synthesis machinery [38].

### Histone methylation

Methionine is a well-known precursor of s-adenosylmethionine (SAM) [10], and, in turn, SAM is constantly required for multiple biological processes, including transsulfuration, polyamine biosynthesis, DNA methylation [15], and histone methylation [39]. The latter is one of the main mechanisms by which the genetic information contained in the DNA is made available (i.e., chromatin status) for transcription and translation into proteins. Within the context of dairy cattle, Bionaz and collaborators [40] observed marked alterations associated with the chromatin status (i.e., euchromatin or available DNA) in bovine mammary tissue from late pregnancy to lactation. Thus, this underscores the importance of understanding the impact of Met availability on histone methylation and its consequences on gene expression and mammary gland biology not just in dairy cattle but in mammals in general.

The results obtained via FRET technology in this study confirmed that Met could alter the histone methylation in H3K9 and H3K27 in immortalized bovine mammary cells (Fig 1A and 1C). However, there was a contrasting methylation effect between H3K9 and H3K27, while H3K9 methylation described a more evident pattern from 0 to 24 h with greater methylation at 125 μM Met, the methylation in H3K27 was more scattered over time (Fig 1C). This contrasting effect was confirmed by FRET analysis at 24 h only (Fig 2), where maximal methylation of H3K9 and H3K27 was observed with 125 μM, and 250 μM Met, respectively. The lack of a dose-dependent response on H3K9 and HK27 methylation suggests that additional mechanisms are likely to play a role in the control of histone methylation. Because this is the first study evaluating the effects of Met (or methyl donors) on histone methylation in bovine, this reduces the ability to assess and extrapolate the magnitude of our results in the context of the dairy cow physiology. The lack of explicit evidence on the effects of Met on histone methylation can be extended to other mammalian models, where this effect has been very seldom interrogated. For instance, Esfandiari and collaborators [41] observed a decline in histone methylation (i.e., H3K9me3) when mice were fed a diet containing ethanol, which predictably lowers SAM levels by inhibiting the transmethylation of homocysteine [42]. The latter suggests that Met deficient diets may cause a similar effect observed by Esfandiari, Medici [41] since Met is a precursor of SAM. Findings in Dobosy, Fu [43] partially confirm this effect, where a lower H3K9me2 was observed in mice fed a choline and Met deficient diet. In terms of H3K27, there is less direct evidence that methyl donors such as Met can cause a change in methylation status in H3K27. Momparler, Cote [44] observed a decrease in H3K27 methylation when cancer cells were incubated with Met metabolism inhibitors. In bovine, H3K27me3 has been mostly evaluated in embryonic development [45, 46], and, in bovine lymphocytes, H3K27m3 was associated with gene silencing [8]. The same pattern was observed in dairy cows during subclinical mastitis, where gene silencing was attributed to H3K27me3 [9].

The biological significance of histone methylation through Met supply in bovine mammary cells and how this molecular process can affect the overall milk biosynthesis in dairy cows remains unclear. The more evident effect of Met on H3K9 methylation put forth the idea of this lysine residue to be a potential candidate for nutriepigenomic effects via dietary Met or methyl donors in the future.

The role of *SUV39H1* and *SETDB1* as histone methyltransferases is well known, especially for the H3K9 [47, 48]. Although there is a lack of data on the direct connection between dietary Met and these histone methyltransferases, this connection is logical since Met serves as methyl-donor for SAM. Silencing the transcription of MATIIα (encoded by *MAT2A*), one of the two isoenzymes that catalyze the synthesis of SAM from Met, suppressed H3K9me3 formation. This effect was associated with the activity of SETDB1 but not SUV39H1 for certain specific genes, such as *Ptgs2* [49]. Kinetics studies have demonstrated the affinity of SUV39H1 to utilize SAM and methylate H3K9 [50]. In the current study, it could be speculated that the linear increase in *SETDB1* was influenced in part by the linear increase in Met supply. Interestingly, the negative quadratic effect *SUV39H1* was contrasting with the positive quadratic effect in H3K9 methylation and total protein synthesis, which might suggest a regulatory feedback mechanism by H3K9 methylation. Taken together, these data suggest that there is a connection between Met availability and transcription of histone methyltransferases, which has been confirmed in other biological models; however, it remains to elucidate the contrasting effect of Met between H3K9 methylation and *SUV39H1* in the bovine.

### Global DNA methylation

The importance of DNA methylation in the context of epigenetics has been underscored in ruminants [51] and non-ruminants [52]. The influence of dietary methyl donors on DNA methylation is only beginning to be understood. DNA methylation patterns can occur at global or region-specific DNA methylation levels [53]. In various physiological models, including cancer and atherosclerosis, it has been observed that global and region-specific DNA methylation do not necessarily agreement; for instance, in tumor cells, global hypomethylation can be accompanied by region-specific hypermethylation [53]. The specific mechanisms that determine global and region-specific DNA methylation are yet to be clearly understood. In the context of dairy cows, region-specific DNA methylation has been attributed to gene silencing of milk protein genes such as *CSN1S1* (αS1-casein) in mammary tissue of pubertal heifers or culled non-lactating cows when compared to lactating cows [54]. Dietary methyl donors such as Met have been observed to cause a global DNA hypomethylation in hepatic DNA from peripartal dairy cows [10], and this effect was corroborated by findings in mice fed betaine, a methyl donor, which experienced a similar hepatic global DNA hypomethylation [55, 56]. In the current study, a parallel effect was observed, where global DNA methylation was gradually reduced as greater concentrations of Met were supplied in the media of bovine mammary cells (Fig 3A). Although the latter confirms the results observed in peripartal dairy cows [10] and rodents [55], it does not explain the underlying mechanism by which a greater substrate of methyl donors may lead to lower global DNA methylation. Previously, James, Melnyk [57] indicated that SAHH might play a role in DNA methylation by inhibiting methyltransferases, specifically DNA methyltransferases. Then, it is conceivable that a similar effect occurs in the bovine model, where SAHH suppressed DNA methylation as a feedback mechanism to restrict an otherwise uncontrollably hypermethylation under a surplus of methyl groups. Interestingly, the latter effect could help explain the global DNA hypomethylation in the current study. The linear upregulation in *SAHH* (Fig 4) was accompanied by a linear downregulation in *DNMT3A*, and a negative quadratic effect in *DNMT1* (Table 1). This makes a plausible case for an inhibitory mechanism of SAHH on DNA methylation in the bovine genome.

The interactions between histone methylation and DNA methylation have been reported [58]. For instance, ubiquitin-like proteins associated with the cell cycle can have a higher affinity to bind H3K9me3 (trimethylated) when its nucleosome DNA contains a higher degree of methylated CpG [59]. Additionally, others observed inhibition of DNA methylation in the presence of H3K27me3, while increased levels were found with H3K9me3 [58]. Our data do not indicate a strong correlation between histone and DNA methylation since a linear decrease was observed in DNA methylation, while a quadratic increase was observed in H3K9 and H3K27.

## Conclusions

**Fig 5:**
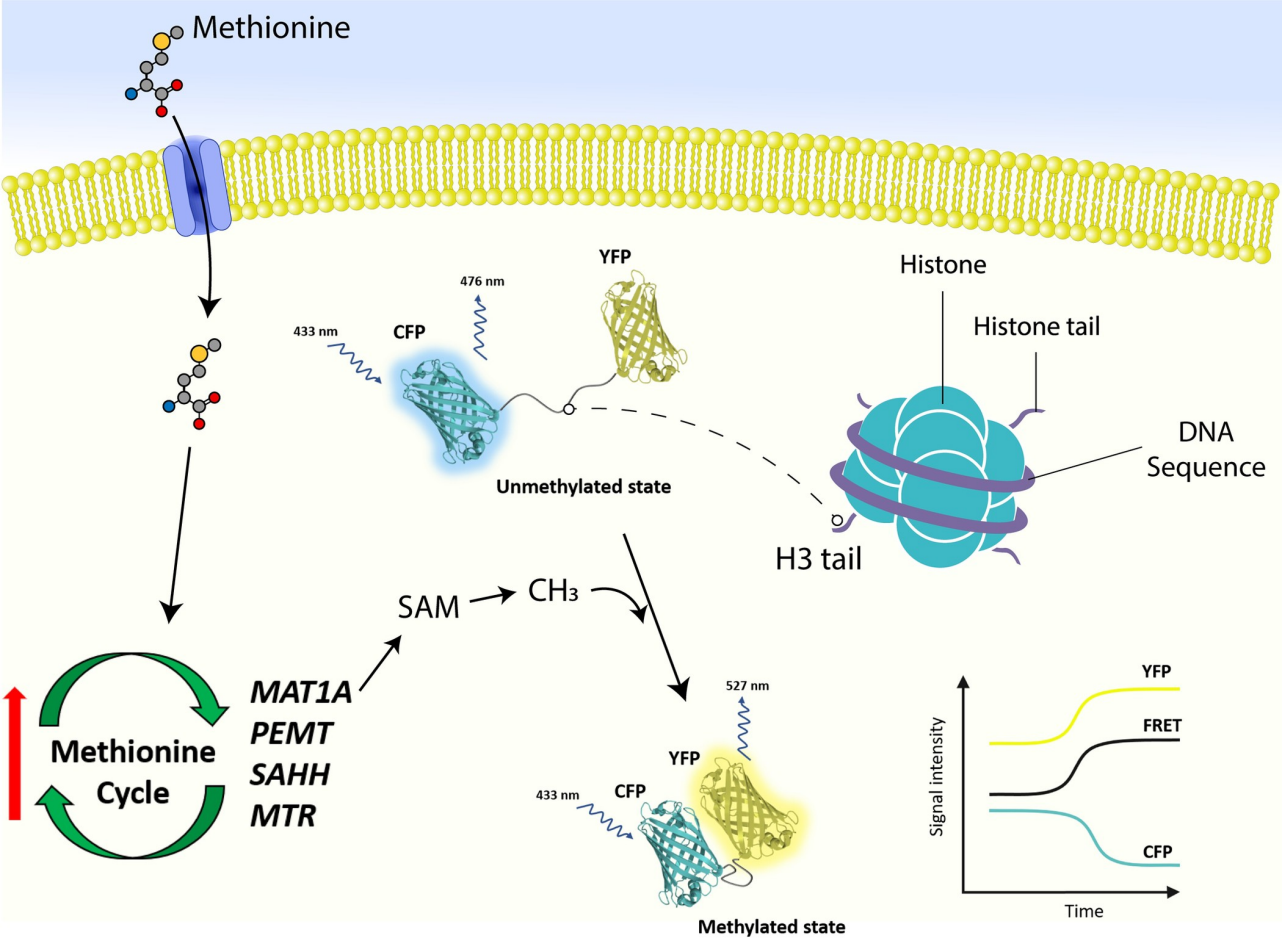
Schematic representation of the Met supply effect on the Met cycle and subsequent histone methylation analyzed via a genetically encoded fluorescent reporter of histone methylation. The influx of Met into the cell will cause an upregulation of genes encoding for enzymes related to the Met cycle, including MAT1A, PEMT, SAHH, and MTR. MAT1A is directly responsible for the conversion of Met into SAM, which is the main methyl donor in the body. This is utilized to methylate DNA and histones, and in this study, the fluorescent reporter was used to assess histone methylation levels. This reporter is composed of a peptide domain replica of the histone 3 tail (H3 tail) encoding either the lysine 9 (H3K9) or lysine 27 (H3K27) positions, which is joined to a methyl-lysine binding domain. This fusion is constructed between a fluorescent resonance energy transfer (FRET) system composed of a dual-fluorescent protein system: cyan fluorescent protein (CFP) and yellow fluorescent protein (YFP). An unmethylated state of the FRET results in high fluorescence emission (476 nm) from the donor (CFP) and low or zero excitation from the receptor (YFP). Upon methylation, the methyl-lysine binding domain in the reporter undergoes conformational rearrangement, increasing the proximity between CFP and YFP, thus, increasing the energy transfer (or FRET) from CFP to YFP. This, consequently, increase the fluorescence emission (527 nm) of the receptor YFP. The ratio of total intensity of CFP-YFP over the CFP was calculated to obtain a YFP signal, which is the signal associated with histone methylation.

The utilization of fluorescent protein systems for real-time imaging of cellular processes coupled with derived quantitative data from such images using pattern recognition software is an untapped technology in gene regulation research from a nutritional standpoint. Our results provide evidence for the potential of this technique for future applications not only on nutriepigenomics but also for other types of nutrient-gene interactions or cellular processes such as nutrient uptake, intracellular bioavailability, energy status (i.e., ATP), among others. The histone methylation data suggest that, to some extent, methyl-donors such as Met may affect the methylation sites, H3K9 and H3K27, and consequently causing a different epigenetic alteration (Fig 5). Gene expression data confirmed that genes related to the Met cycle are highly responsive to Met supply, and the upregulation of *SAHH* may be associated with a decline in DNA methylation, as previously observed in other biological models. In the context of the dairy cow, further refinement to this FRET assay to study histone methylation could lead to establishing novel potential mechanisms of how dietary methyl donors may control the structural conformation of the bovine genome and, by extension, gene expression.

## Supporting information

S1 File (DOCX)
