# Supplementary material for: Quantitative determination of histone methylation via fluorescence resonance energy transfer (FRET) technology in immortalized bovine mammary alveolar epithelial cells supplemented with methionine
Source: PLoS One. 2020 Dec 21;15(12):e0244135. doi: 10.1371/journal.pone.0244135 (PMC7751961; doi:10.1371/journal.pone.0244135)
Supplement: S1 File — (DOCX) [file pone.0244135.s001.docx]

**S1 FILE**

**Design and evaluation of primers**

Primers were designed using Primer Express (version 2.0 or 3.0) with min amplicon size of 80 bp (but when possible were preferred amplicon ≥ 100 bp) and limited 3’ G+C (Applied Biosystems, CA). Major part of the primers sets were designed to fall across exon–exon junctions. Primers were aligned against publicly available databases using BLASTN at NCBI and UCSC’s Cow (*Bos taurus*) Genome Browser Gateway (http://genome.ucsc.edu/cgi-bin/hgGateway).

Prior to perform qPCR, the primers were tested in a 20 μL PCR reaction using the same protocol described for qPCR except the final dissociation protocol. For this purpose, we used a universal reference cDNA (RNA mixture from 5 different tissues) to be sure to identify the gene. Five μL of the PCR product was run in a 2% agarose gel stained with ethidium bromide. The remaining 15 μL were cleaned using QIAquick^®^ PCR Purification Kit (QIAGEN) and sequenced at the Genomic Center at University of Minnesota. Only primers that presented a single band at the expected size and the right amplification product (verified by sequencing) were used for qPCR. The accuracy of a primer pair was also evaluated by the presence of a unique peak during the dissociation step at the end of qPCR.

**cDNA synthesis**

The cDNA reaction was performed with 100 ng of RNA. The RNA was mixed with the Master Mix-I (MM1) containing 9 µL DNase/RNase free water and l µL random primers (Cat. no. 11; Roche, Mannheim, Germany). The mixture was incubated at 65°C for 5 min. After incubation, the MM1+RNA was kept on ice for at least for 3 min before adding 9 µL of Master Mix-II (MM2). The MM2 contained the following ingredients: 1.625 µL DNase/RNase free water, 4 µL 5X First-Strand Buffer (shipped with RevertAid enzyme (Fermentas), 1 µL Oligo dT18, 2 µL 10 mM dNTP mix (10 mM; Cat. No. 18427-088; Invitrogen), 0.25 µL of Revert aid (200 U/µL; Cat. No. EP 0442; Fermentas), and 0.125 µL of RNase inhibitor (40 U/μL; Cat. No. EO 0382; Fermentas). The final 20 µL vol (MM1+RNA and MM2) was incubated using the following temperature program: 25°C for 5 min, 42°C for 60 min and 70°C for 5 min. followed by 4°C.

**Polymerase Chain Reaction (PCR)**

The resulting cDNA was diluted (1:4) with DNase/RNase free water. After proper vortexing, a 4 µL diluted cDNA sample or standard curve dilution was carefully pipetted in triplicate into MicroAmp™ Optical 384-Well Reaction Plates. A 6 µL of SYBR Green Master Mix composed of the following ingredients was added to each well: 5 µL 1x SYBR Green (Power SybrGreen Master Mix), 0.4 µL of 10 µM forward primer, 0.4 µL of 10 µM reverse primer, and 0.2 µL DNase/RNase free water. The reactions was performed in an QuantStudio 6 Flex Real-Time PCR System (Applied Biosystems, Waltham, MA) using the following conditions: 2 min at 50°C, 10 min at 95°C, 40 cycles of 15 s at 95°C and 1 min at 60°C. The specificity of the amplicons was verified with the dissociation protocol: 95°C for 15 s plus 65°C for 15 s. The data obtained were analyzed using the QuantStudio^TM^ Real-Time PCR Software (version 1.3, Applied Biosystems).
